# Insights into the behavior of six rationally designed peptides based on *Escherichia coli*’s OmpA at the water-dodecane interface

**DOI:** 10.1371/journal.pone.0223670

**Published:** 2019-10-10

**Authors:** Miguel Fernández-Niño, Lina Rojas, Javier Cifuentes, Rodrigo Torres, Andrea Ordoñez, Juan C. Cruz, Edgar Francisco Vargas, Diego Pradilla, Oscar Álvarez Solano, Andrés González Barrios

**Affiliations:** 1 Grupo de Diseño de Productos y Procesos (GDPP), Department of Chemical and Food Engineering, Universidad de los Andes, Bogotá, Colombia; 2 GINIB Research Group, Department of Biomedical Engineering, Universidad de los Andes, Bogotá, Colombia; 3 Grupo de Investigación en Bioquímica y Microbiología (GIBIM), School of Chemistry, Universidad Industrial de Santander, Bucaramanga, Colombia; 4 Laboratorio de Termodinámica de Soluciones, Department of Chemistry, Universidad de los Andes, Bogotá, Colombia; Massachusetts Institute of Technology, UNITED STATES

## Abstract

The *Escherichia coli*’s membrane protein OmpA has been identified as a potential biosurfactant due to their amphiphilic nature, and their capacity to stabilize emulsions of dodecane in water. In this study, the influence of surfactant type, concentration, preservation time and droplet size on the crystallization of *n*-dodecane and water, in oil-in-water emulsions stabilized with six rationally designed *Escherichia coli*’s OmpA-based peptides was investigated. A differential scanning calorimetry (DSC) protocol was established using emulsions stabilized with Tween 20^®^ and Tween 80^®^. A relationship between the surfactant concentration and the crystallization temperatures of *n*-dodecane and water was observed, where the crystallization temperatures seem to be dependent on the preservation time. A deconvolution analysis shows that the peak morphology possibly depends on the interactions at the interface because the enthalpic contributions of each Gaussian peak remained similar in emulsions stabilized with the same peptide. Adsorption results show that the main driver for adsorption and thus stabilization of emulsions is polar interactions (e.g. H-bonding) through the hydrophilic parts of the peptides. Those peptides with a preponderance of polar interaction groups distribution (i.e. NH_2_, COOH, imidazole) showed the highest interfacial activity under favorable pH conditions. This suggests that custom-made peptides whose hydrophilic/hydrophobic regions can be fine-tuned depending on the application can be easily produced with the additional advantage of their biodegradable nature.

## Introduction

Recently, the scientific community has centered its efforts in the development and identification of biological surfactants to replace chemically derived surfactants due to their ecological acceptability [1]. The relevance of these biosurfactants in different industries such as food additives, cosmetics, enhancement of oil recovery and bioremediation has been extensively highlighted [1–5]. They display different chemical structures depending on the species that are used for their production, which make them more attractive than their conventional hydrocarbon-based counterparts. Thus, they are classified according to their structure in glycolipids, phospholipids, lipopeptides, and polymers biosurfactants [6].

*Escherichia coli’s* transmembrane protein OmpA has drawn attention in the biosurfactant field because it has been found to exhibit surfactant activity thought experimental assays [7]. In fact, we have previously shown the ability of this protein to increase the stability of n-dodecane in water emulsions [7]. In order to further dissect the microscopic features that were related to the stability of emulsions with OmpA, we have also studied the interactions of this protein at the n-dodecane-water interface trough *in silico* molecular dynamics simulations based on free energy calculation during insertion [8]. In addition, we have previously designed sixteen peptides by taking into account the hydrophobic profiles shown in the hydropathy plot of OmpA and properties such as free Gibbs energy normalized with solvent accessible surface area (ΔG_sol_ /SASA) and the molecular weight (ΔG_sol_ /MW), obtained from *in silico* analysis [8]. This ability to rational design peptides through molecular dynamics simulations provide many possibilities in terms of functionality, three-dimensional structure and performance at liquid-liquid interfaces and liquid-solid surfaces [8–10]. Nevertheless, the overall quality of the simulated properties of a molecular system will rely on (i) the accuracy of the interatomic interaction function and (ii) the degree of sampling and convergence reached in the simulation [11]. Besides, this type of simulations do not account the intermolecular interactions of surfactant molecules when are arranged in supramolecular structures such as micelles, and more importantly, they do not contemplate the evolution of dynamic properties like droplet size distribution and polydispersity [8]. This information is critical in order to maintain the functionality of the formulation. In this regard, the present work aimed to further study the thermodynamic and dynamic behavior of six rationally designed peptides in *n-*dodecane-in-water emulsions, via Differential Scanning Calorimetry (DSC), Dynamic Light Scattering (DLS) and Interfacial Tension (IFT) measurements. Here, the top six peptides highlighted in our previous study through molecular dynamic [8] were synthesized via solid-phase peptide synthesis and O/W emulsion were prepare mimicking the proportion of the species in the simulation. Samples were thermally treated to assess the effect of surfactant type, surfactant concentration and droplet size distribution on the liquid to solid transitions of n-Dodecane and water in the emulsion. Furthermore, interfacial tension measurements were conducted in order to compare the DSC and DLS results with observations on the adsorption behavior at the crude oil-NaCl 1M interface.

## Materials and methods

### Emulsion formulation and emulsification process

Lyophilized peptides were synthesized via rapid solid-phase following standard protocols [12]. The sequence, length, charge, isoelectric point and molecular weight of the analyzed peptides are shown in Table 1. As a starting point, stock solutions of peptides were prepared in 500 μl of deionized water. Subsequently, emulsions at 0.05%, 0.15% and 0.25% (w/v) of surfactant (i.e. peptide) were obtained in a two-step process. The samples used to study the influence of the preservation time and to measure the droplet size distribution were prepared with 0.25% (w/v) of the peptide. In the first step, the surfactant stock solution was mixed for 5 minutes with water to complete 627μL in a 750 W ultrasonic processor (VC 750, Sonics and Materials Inc., Newtown, Connecticut, USA). The homogenization was executed using pulses with 36% of amplitude for the 40s followed by a pause of 20s. In the second step, *n-*dodecane (Sigma-Aldrich, Missouri, USA) was added to complete 1mL of emulsion (373μL) and mixed for 20 minutes using the same procedure.

**Table 1:** Sequences of the six peptides that were synthesized with their length, molecular weight (MW), isoelectric point (pI) and charge (Ch).

Peptide	Sequence	Ch	MW (g mol^-1^)	pI	Length	Reference
1	*GKNHDTGVSPVFA*	0	1.3	6.74	13	This study
2	*DPKDGSVVVL*	-1	1.1	4.21	10	This study
3	*TGNTCDNVKQR*	+1	1.2	7.89	11	This study
4	*THENQLGAGAFG*	-1	1.2	5.21	12	This study
5	*QRAALIDCLAPDRRV*	+1	1.7	8.25	15	This study
6	*QRAALIDCLA*	0	1.1	5.83	10	This study
**-**	OmpA	-5	35	5.60	325	[8]

### Biosurfactant effect on the transition temperature for both n-dodecane and water phases

In this research, a calorimetric analysis was carried out using a heat flux Differential Scanning calorimeter (DSC) Q2000 (Thermal Analysis (TA) Instruments, New Castle DE). A graph of heat flow vs. temperature (thermogram) was obtained where peaks represent the changes in heat capacity due to a thermal transition. In other words, the obtained endothermic peaks can be associated with melting phenomena, while exothermic peaks are associated with crystallization. Before establishing the baseline, the cell’s constant and temperature were calibrated using Indium (Sigma-Aldrich, Missouri, USA). The samples were prepared right after homogenization (i.e. after 2 minutes) in aluminum hermetic capsules with a sample size of 5–15 mg. The calorimeter conditions were: 1) equilibration step at 60°C; 2) cooling to -50°C at a rate of 5°C min^-1^; 3) isothermal for 5 minutes and 4) heating to 60°C at a rate of 5°C min^-1^. At least three replicates of each emulsion were analyzed using freshly prepared samples. A mixture of *n-*dodecane-water (5:1) was used as a negative control. The samples used to study the evolution over time were positioned into hermetic capsules 2 minutes after homogenization and kept at room temperature for two and four hours before the thermal treatment.

### Analysis of differential scanning calorimetry data

Onset temperatures were obtained as the local maximum in temperature vs. time plot and enthalpies were calculated using the software Universal Analysis 2000 from TA Instruments. All the composed peaks in the thermogram were deconvolved into a series of Gaussian peaks using the OriginPro v7.5 software (Origin Lab Corporation, USA). Thus, it was possible to obtain information about the thermodynamic parameters of thermal transitions. These parameters that could change with crystallization can be quantified and placed into the Avrami equation to measure crystallization rates. Thus, the ratio between the ordinates of a DSC curve and the total area of the peaks was used to determine the corresponding crystallization rates. Crystallization is a complex process consisting of two major events: nucleation and crystal growth. The curves were fitted with the Levenberg Marquardt algorithm using the full width at half maximum (FWHM) version of a Gaussian function.

### Droplet size measurement

The average droplet size was measured with the dynamic light scattering (DLS) instrument Zetasizer NanoZS (Malvern Instruments, Worcestershire, UK), at λ = 633 nm, temperature = 25 °C and angle = 173°. The droplet size was registered at zero, two and four hours after emulsion preparation with 0.25% (w/v) of surfactant.

### Interfacial tension measurement

A mixture of crude oil and n-dodecane (25% (w/v)) was initially prepared, subsequently stirred for 40 minutes at 2000 rpm using a high-speed mixer Dispermat (VMA-Getzmann GmbH, Germany) and degasified for 30 minutes in a Bransonic 2510 ultrasonic bath (Branson Ultrasonics, USA). Then, samples of 2 mL composed of diluted crude oil and peptide (at a final concentration of 550 ppm) were homogenized in a 750 W ultrasonic processor (Sonics and Materials Inc., USA) for 5 minutes (19% of amplitude and pulse of 1 s on followed by 1 s off). The interfacial tension (mN/m) of these samples was then determined at pH 7 using an Attension tensiometer (Biolin ScientificTM, Sweden) following the Young Laplace method [13]. All measurements were carried out at room temperature (21± 1°C) for 300 s, and at least three replicates were performed for each sample. As a negative control, a sample containing only the diluted crude oil was measured under the same conditions. The crude oil used for interfacial tension measurements was previously characterized (S2 Table) by the SARA Assay as proposed by Liyana *et al*. (2014) [14]

### Critical micelle concentration

The critical micelle concentration (CMC) was defined as the concentration of surfactant above individual molecules aggregates into micelles, which represents a threshold between individual molecules of surfactant and surfactant micelles in a dynamic equilibrium [15]. The CMC of surfactant solutions was determined using a Nano Isothermal Titration Calorimeter (Nano ITC TA instruments). The experiments were performed at 25 °C, using 300 μL of water into the sample cell and titrated with a solution of OmpA (0.109 mg·mL-1) solution by means of titration syringe. The system was left to stabilize 120 min and then the injections were made. The software TA NanoAnalyzeTM was used to analyze the obtained ITC data [16]. In addition, the CMC was also measured using interfacial tension using a tensiometer (DCAT Dataphysics) and applying the Wilhelmy plate method [17]. The experimental procedure was similar to that used in Nano ITC.

### Peptides secondary structures analysis

Secondary structures of peptides were studied by analysis of second derivative amide I Fourier Transform Infrared spectra. Infrared spectra were recorded using an A250/D FT-IR (Bruker, Germany). Peptides were solubilized in type I water, and then, spectra were recorded in the range of 4000–400 cm−1 with a spectral resolution of 2 cm−1. The water infrared spectrum was digitally subtracted to avoid the interference of water absorbance in the range of 1700–1600 cm−1 related to the H-O-H bending [1]. Next, the second derivate of the infrared spectra in the range of amide I band (1700–1600 cm−1) were calculated. Finally, the different peaks present in the secondary derivates were associated with a specific secondary structure element based on previously reported data [18–20].

## Results and discussion

### Peptide type, concentration and crystallization temperatures

In this work, the behavior of six rationally designed peptides at the oil in water interface was first studied by means of variations in the crystallization phenomenon in oil-in-water (O/W) emulsions. Several reports have shown that surfactants may induce heterogeneous nucleation at the interface in oil-in-water emulsions, which influence particles formation after individual crystallization of organic droplets [21–26]. In the particular case of O/W emulsion systems, several factors can affect crystallization [27][28]. For instance, it has been previously shown that crystallization temperatures of water and n-dodecane are affected by the amount and type of surfactant in the emulsion [21,22][29,30].

**Fig 1:**
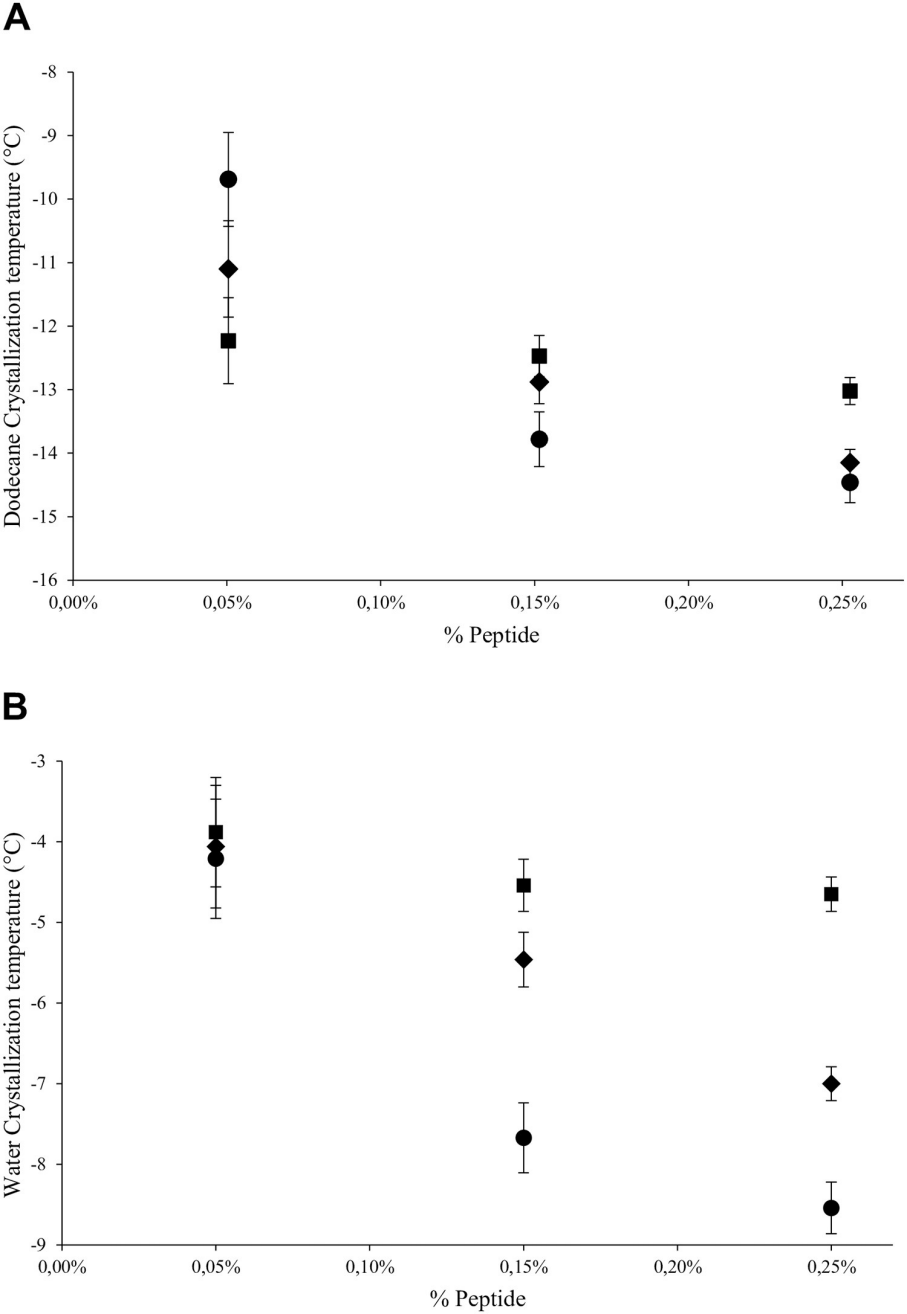
Influence of peptide concentration on the crystallization temperature (°C) of water (A) and n-dodecane (B) in O/W emulsions. Circles, rhombus, and squares represent peptides 1 (GKNHDTGVSPVFA), 2 (DPKDGSVVVL) and 5 (QRAALIDCLAPDRRV) respectively.

Here, a DSC assay was initially performed to study the effects of surfactant concentration and surfactant type on the interactions that take place at the oil-water interface. This approach has been previously used to characterize complex W/O emulsions from the oil industry [31][32,33]. Our data revealed that only those emulsions stabilized with the peptides 1, 2 and 5 showed an inverse correlation between the amount of surfactant and the crystallization temperature of both water and *n-*dodecane (Fig 1 and S1 Fig). The observed correlation could be attributed to a surface coverage problem and the density of the adsorbed layer in which not every droplet in the emulsion is homogeneously covered by the surfactant. In order to determine the concentration required to coat these droplets, the critical micellar concentration for OmpA was then determined by ITC and surface tension measurements. This value was between 1.2*x*10^−3^ and 2.7*x*10^−3^ mg mL^-1^, which indeed is in the same order of magnitude of the peptide counterparts. As a consequence of the smaller molecular size of the designed peptides as compared to traditional surfactants for O/W emulsions (e.g. Tween 20 (MW = 1228 g/mol)), it can be concluded that these peptides will form irregular molecular aggregates, where molecular interactions between them will dominate the shape and places where the agglomeration process will occur [34]. This suggests that the effect on the transition temperature might be attributed to other additional factors such as differences in the free energy change during insertion at the *n-*dodecane-water interface. In fact, our previous molecular dynamics simulations [9] have revealed that peptides 1, 2 and 5 displayed the highest free energy change per molecular weight (ΔG_solv_/MW) thus suggesting that the effect on the transition temperature is also associated to the energy during insertion (S1 Table).

As shown in S2 Fig, cooling thermograms for all the peptides at 0.05%, 0.15% and 0.25% (w/v) were also obtained. The shape of the peaks for the same peptide was shown to be maintained for all tested peptide concentrations, which is presumably due to similar interactions that take place at the oil-water interface. Then, in order to further study those peaks, a deconvolution analysis was carried out to find connections between the peak morphology and particular interactions that could happen at the oil-water interface. Our data revealed that the enthalpy contributions of each Gaussian peak were stable in all the emulsions stabilized with the same peptide at different concentrations. This might be indicative of the relationship between the shape of the peaks and the interactions of the surfactant at the oil-water interface.

**Fig 2:**
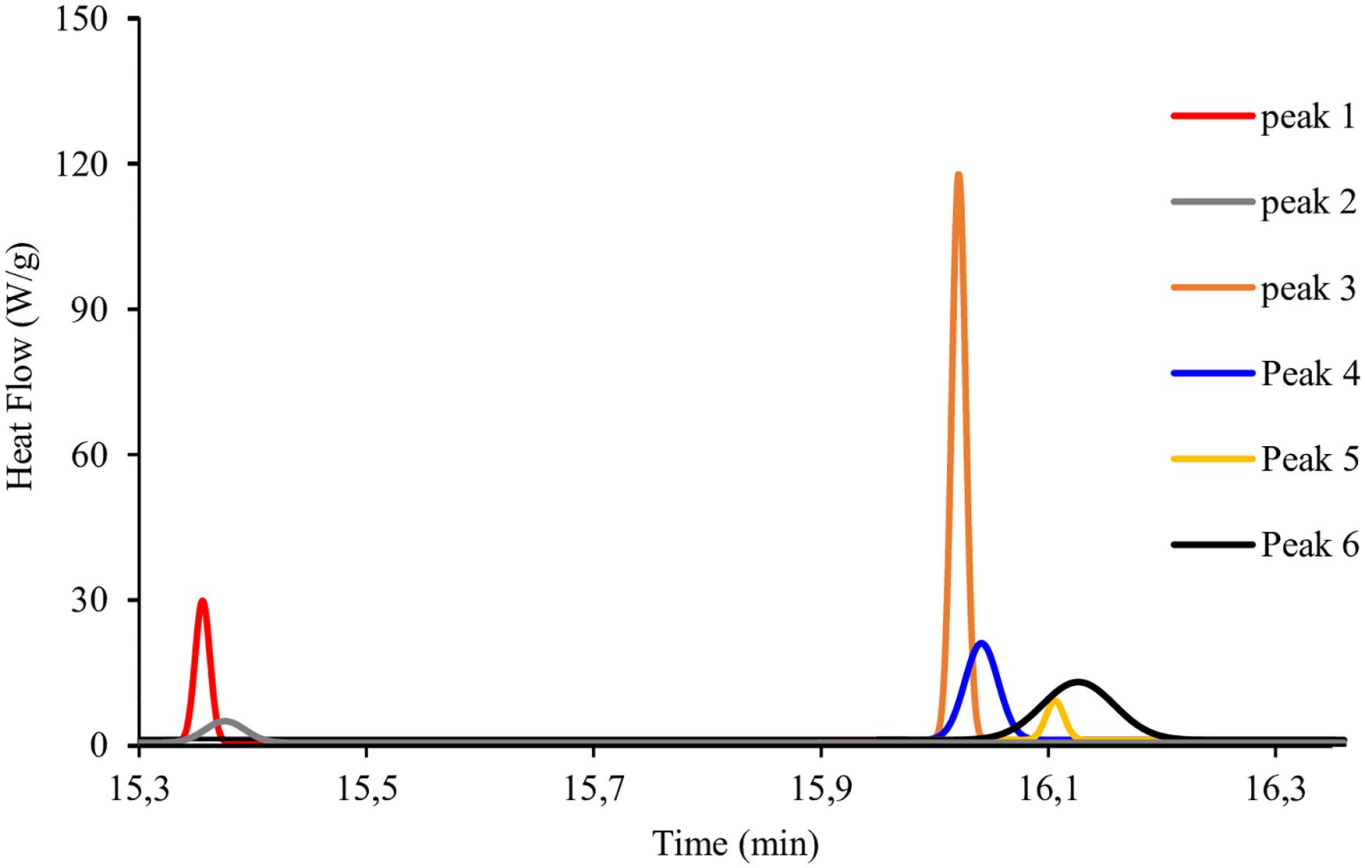
Gaussian peaks. Associated with peak 1: nucleation of dodecane, peak 2: growing crystal of dodecane, peak 3: nucleation of water, peak 4: growing crystal of dodecane, peak 5: nucleation of the remaining dodecane, peak 6: growing crystal of the remaining dodecane.

Crystallization is a two-stage process involving the formation of nuclei followed by crystal growth [35]. According to Lorenzo and Müller [36], if the overall crystallization kinetics is determined by differential scanning calorimetry, both primary nucleation and crystal growth will make a contribution resulting in a superposition of both bell-shaped curves. Thus, it was determined that the cluster of crystallization peaks was composed of six sub-peaks: a peak attributed to the nucleation of *n-*dodecane, a peak related to the growth of *n-*dodecane crystals, a third and fourth peak attributed to nucleation and crystallization of water respectively and a fifth and sixth peak associated with the final crystallization of the remaining liquid droplets of *n-*dodecane (Fig 2).

**Fig 3:**
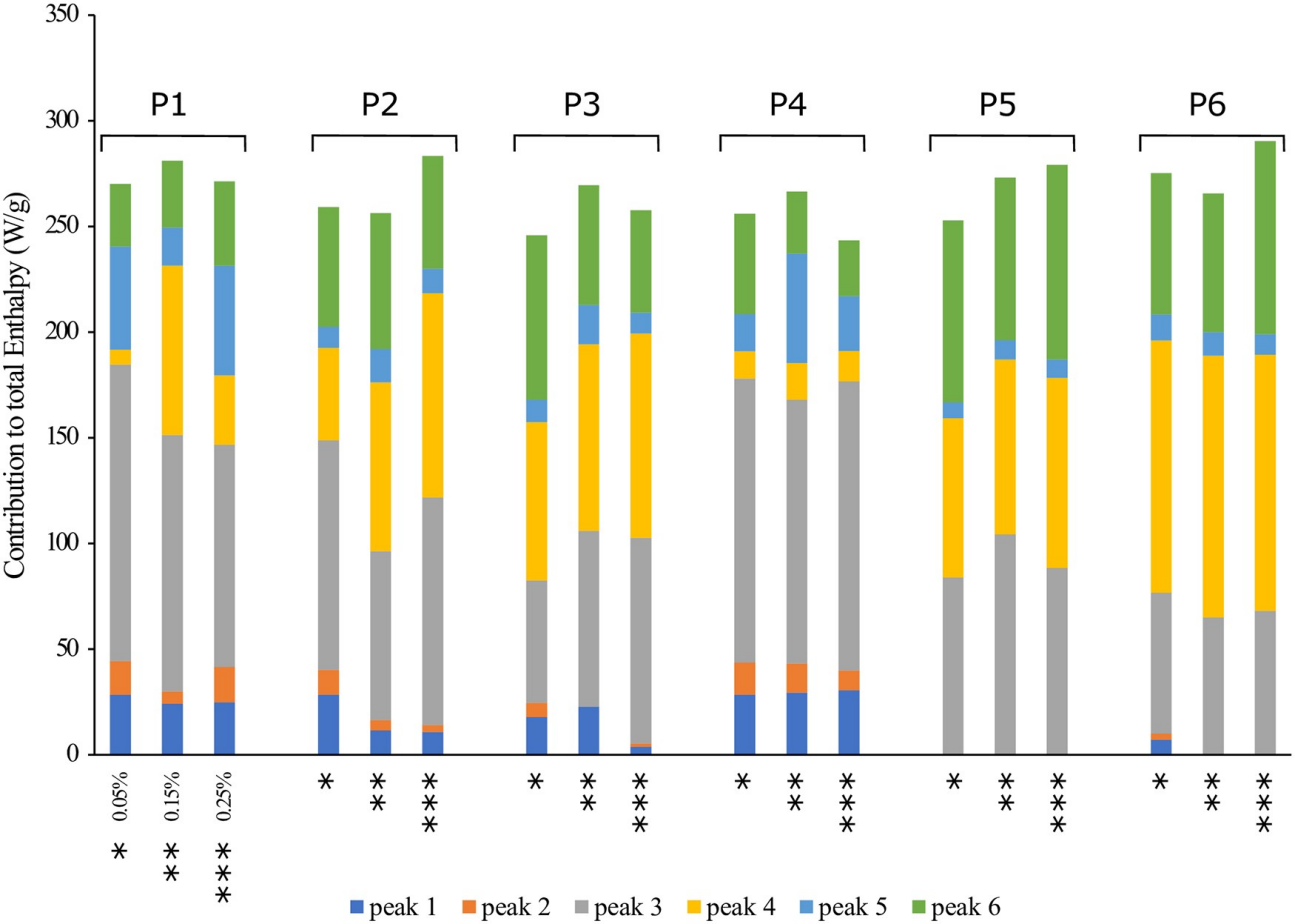
Enthalpy contribution of each Gaussian peak to the total crystallization enthalpy. Including n-Dodecane and water for 0.05%, 0.15% and 0.25% (w/v) of the peptide in the emulsion.

**Fig 4:**
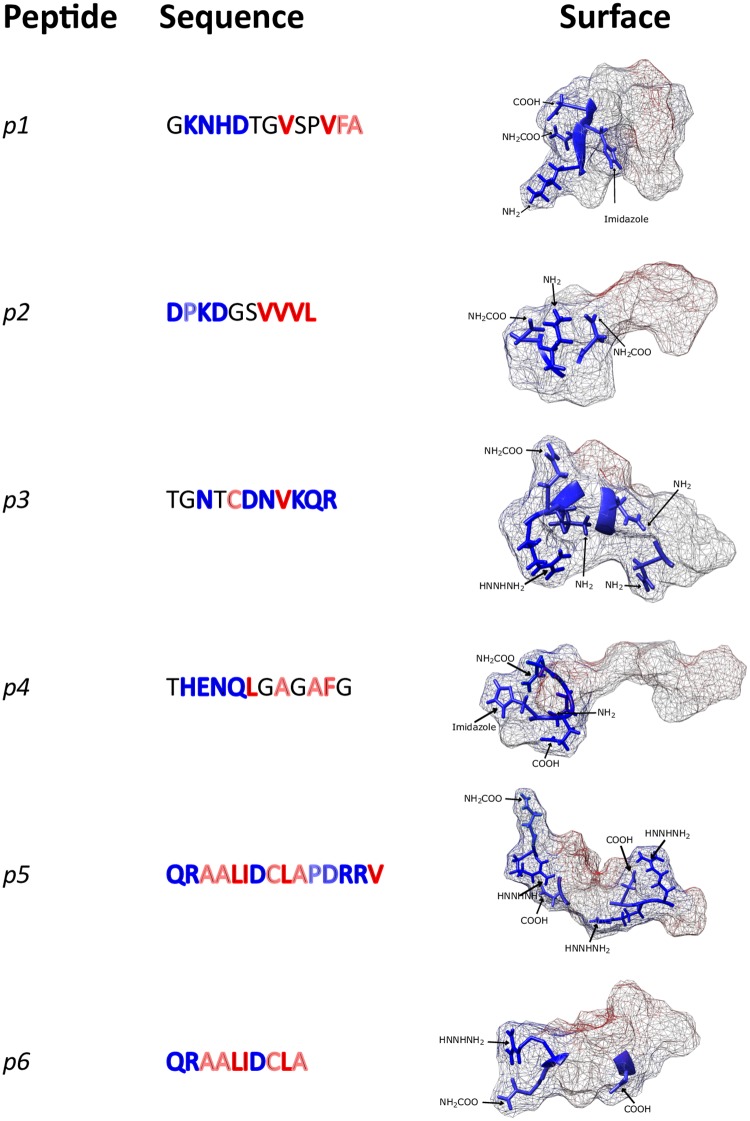
Structural patterns of hydrophilic (blue) and hydrophobic (red) residues and surface simulation for the six synthesized peptides.

Further evaluation of the enthalpy of each Gaussian peak revealed that the contribution to the total enthalpy remains similar for all the emulsions stabilized with the same peptide regardless of the concentration (Fig 3). Also, the emulsions stabilized with peptides 5 and 6 did not show the first two peaks. These results suggest that nucleation and formation of *n-*dodecane crystals do not play a significant role in the enthalpy change for peptides with low free energy change per hydrophobic area. Moreover, considering the amino acids present in both peptides (Gln-Arg-Ala-Ala-Leu-Ile-Asp-Cys-Leu-Ala), the non-continuous distribution of the hydrophilic amino acids can be associated with hydrophobic moieties delimitated by two hydrophilic moieties for both peptides (Fig 4). This structural feature could give them a dissimilar configuration at the oil-water interface when compared to the other peptides.

It was also observed that peptide 4 and peptide 1 showed a similar enthalpy distribution among the set of peptides (Fig 3). Analyzing the amphiphilic nature of those two peptides, in both structures, all the ionizable residues remained in the hydrophilic head: Lys, Asn, His and Asp for peptide 1, and His, Glu, Asn and Gln for peptide 4 (Fig 4). This characteristic confers them the ability to ionize in solution which causes the reduction of non-favorable interactions at the liquid-liquid interface. Additionally, the presence of ionizable residues gives these peptides interesting properties, since the electrostatic interactions can be manipulated by varying the pH. It is well known [37] that near the isoelectric point, proteins have both negative and positively charged groups in roughly equal numbers, and so it is possible for adsorption at the oil-water interface to be enhanced by electrostatic interactions. This explains the improved surface coverages of proteins observed at pH values close to their isoelectric points.

### Stability and droplet size

**Fig 5:**
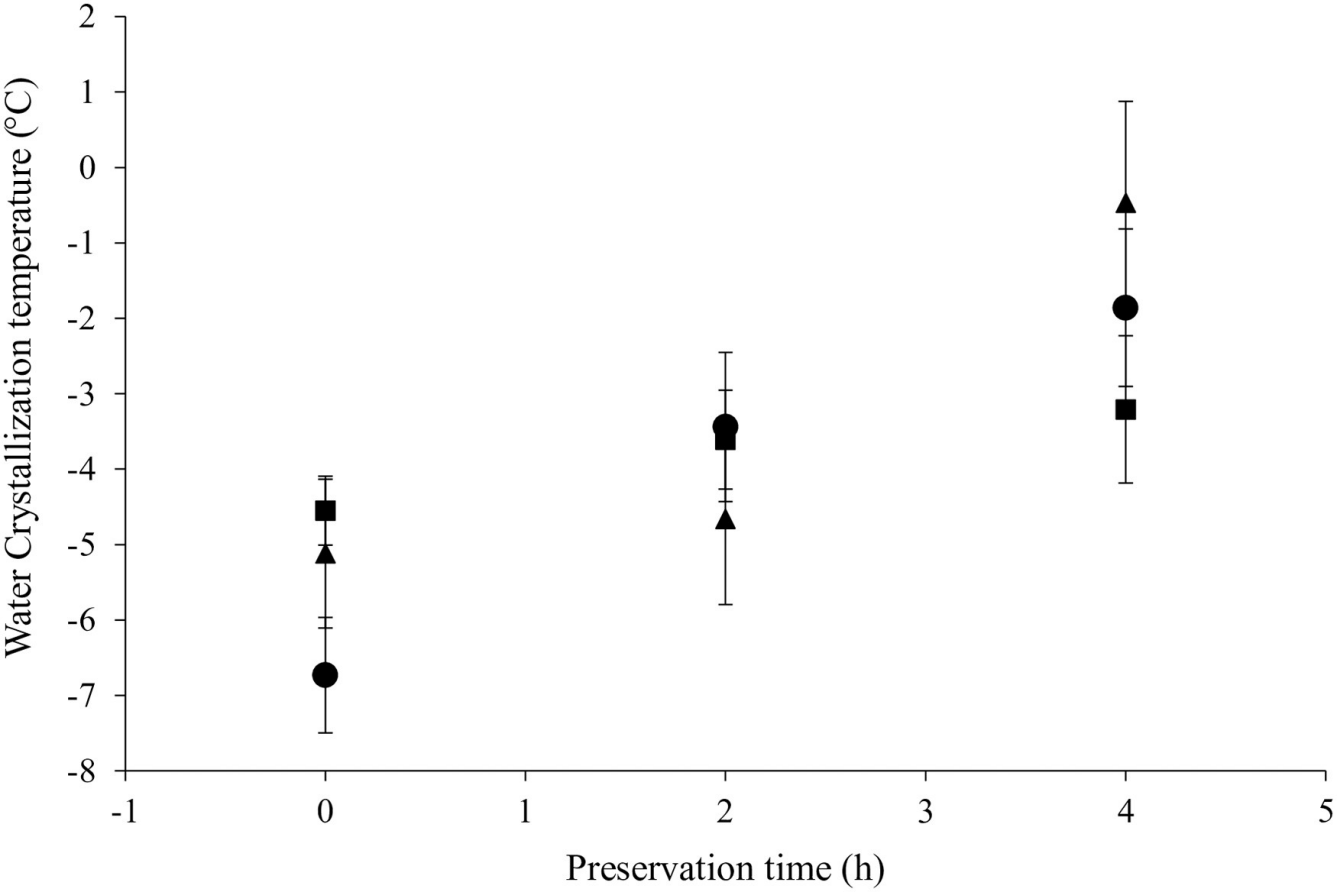
Influence of the preservation time on the water crystallization temperature. Circles, rhombus, and squares represent peptides 1 (GKNHDTGVSPVFA), 4 (THENQLGAGAFG) and 5 (QRAALIDCLAPDRRV), respectively. The peptide 5, which exhibit less variation in the droplet size also showed the lowest variation in the water crystallization temperature.

In order to further study the behavior of the six rationally designed peptides at the oil in water interface, the influence of preservation time and droplet size on the crystallization temperature was investigated. Our data revealed a strong correlation between the preservation time and crystallization temperature only for peptides 1, 4 and 5 (Fig 5 and S3 Fig), which agrees with our previous results regarding the effect of peptide concentration for peptide 1 and 5. It may be possible that these peptides displayed a different behavior as compared to the other peptides due to the fact that they have clear hydrophilic heads with ionizable residues, which allow them to interact electrostatically with water molecules at the interface.

**Fig 6:**
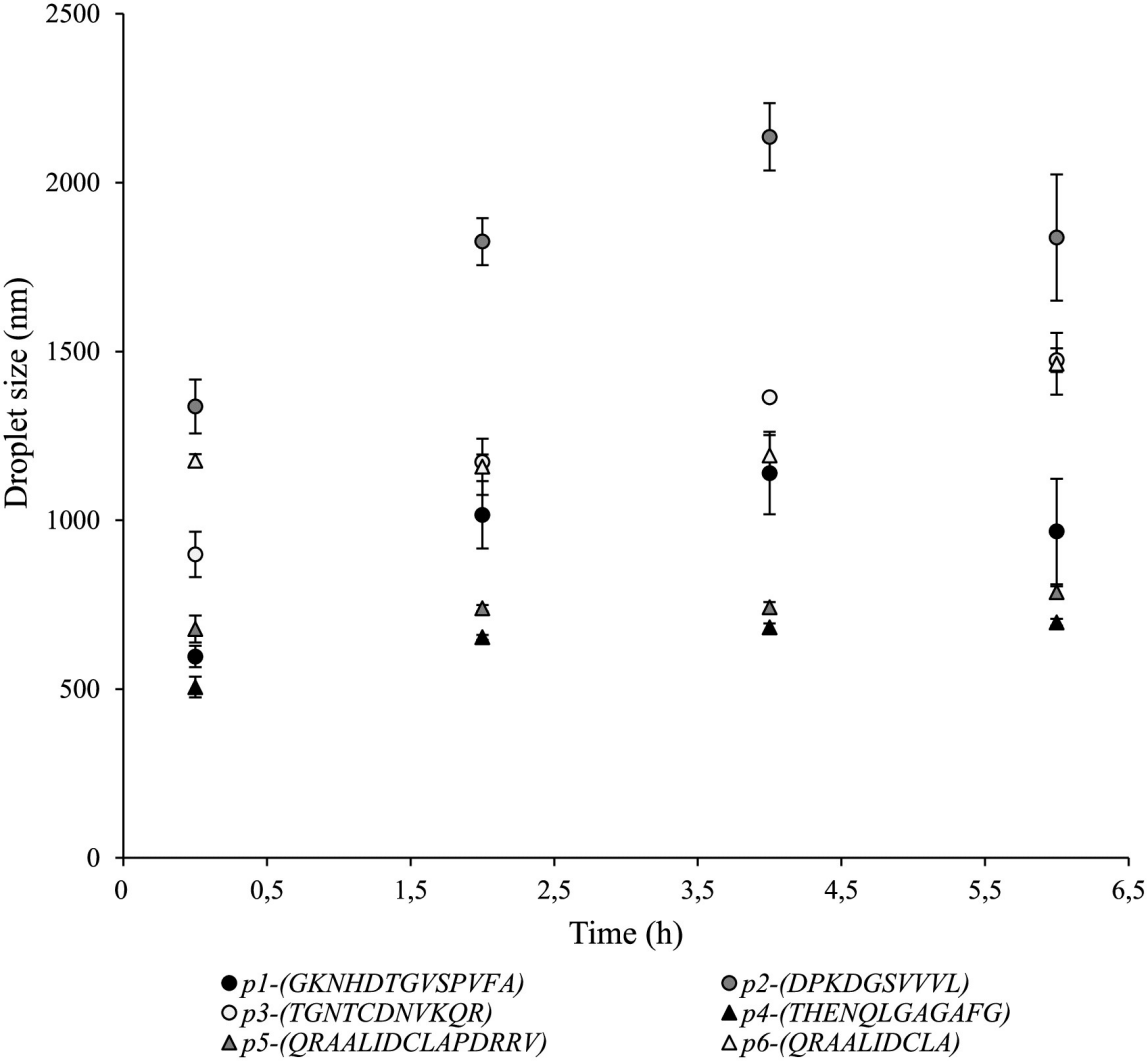
Droplet size distribution for all the peptides measured via Dynamic Light Scattering.

Analyzing the results with regard to the droplet size distribution (Fig 6), peptide 5 was also shown to exhibit the lowest change in droplet size over the six hours of the experiment as compared to the other peptides. This could be explained by the magnitude of the steric and electrostatic repulsion forces among droplets. In fact, this peptide (14 residues) was expected to has a particular conformation at the interface caused by the presence of two hydrophobic domains that may be related to the magnitude of the steric and electrostatic repulsions forces.

It is worth noticing that unlike the other peptides, the droplet size distribution of peptides 1 and 2 decreased at the end of the experiment, which may be related to the fact that the biggest oil droplets in the emulsion became a part of the creamed layer as a result of the flocculation process. However, the high standard deviation suggests that the samples were collected at different heights.

Interestingly, peptide 5, which exhibited less variation in the droplet size distribution over time, also was found to present a lower dependence of crystallization temperature on the preservation time (Fig 5). In contrast, for peptide 4 (that showed a similar tendency in the droplet size distribution over time) the relationship between preservation time and crystallization temperature differed considerably. Therefore, there is no evidence that emulsion stability can be studied by means of changes between the cooling thermogram of emulsions with different preservation times. In this work, it was not possible to link those two variables because the six emulsions with the same concentration of peptide (0.25% (w/v)) behaved differently in terms of phase separation. In other words, each emulsion was found at a different stage of the destabilization process. This is confirmed because phase separation was visually detected by inspection in some of the systems (emulsions stabilized with peptide 3 (*TGNTCDNVKQR*)). However, if the surfactant concentration is set at an interval by which creaming can be avoided, the methodology employed here is suitable to quantitatively assess the destabilization degree during the entire process without suffering the disadvantages of sampling in a multiphase system [33]. Thus, surfactant concentration is crucial to investigate the influence of the surfactant type and preservation time on crystallization behavior. The assessment of destabilization degree inside a hermetic calorimetric capsule seems to be a suitable way to escape the disadvantages of sampling within a two-phase system.

Finally, it is important to mention that the enthalpy distributions for the Gaussian peaks at different preservation times did not present any tendency (S4 Fig), which could be attributed to polydispersity caused by continuous coalescence, caused the appearance of several peaks that were assigned to different droplet sizes [38]. Polydispersity negatively affects the deconvolution analysis and so the fitting procedure showed that the original function was not fully represented by the set of Gaussian peaks.

### Adsorption and liquid-liquid interface

**Fig 7:**
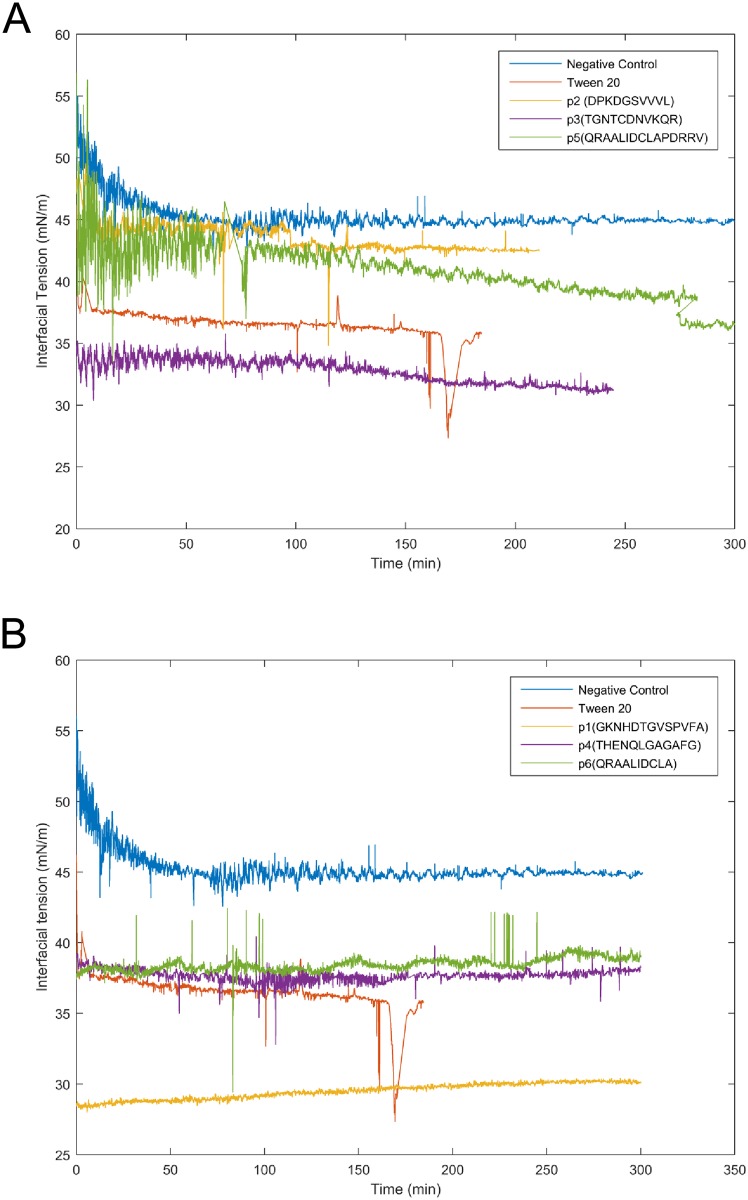
Dynamic interfacial tension for the peptides that (A) take a long time and (B) take a shorter time to reach the interface crude oil-NaCl 1M. All experiments were carried out in triplicates and a representative replicate is shown.

Interfacial tension experiments were performed in order to evaluate the adsorption behavior of the six rationally designed peptides at the crude oil-NaCl 1M interface. Thus, the six rationally designed peptides can be categorized into two different groups according to the mechanism by which they reached the interface in interfacial tension experiments: the peptides that took more time to adopt their configuration with the lowest free energy (Fig 7A) and the ones that adsorbed almost immediately to the interface (Fig 7B). Our data suggest that peptides that reached the interface faster are uncharged or smaller whereas the peptides that took more time to adsorb at the interface are charged or feature a structure without defined hydrophobic and hydrophilic moieties (Figs 4 and 7). It is important to mention that, according to the API scale, the crude oil employed is categorized as heavy. The acidity of the crude oil implies the presence of asphaltenes and resin acids which interact with water and consequently are located in the crude oil-NaCl 1M interphase forming highly stable systems [39]. This can explain why negative control showed a reduction in interfacial tension over time. Regardless of the mechanism by which the peptides reached the interface, in all the cases the oil/water interfacial tension was reduced (Fig 7). Furthermore, the peptide with the lowest free energy of insertion according to the molecular dynamics simulation [8] was the same that reduced to the lowest value the interfacial tension (from 45mN m^-1^ to 30 mN m^-1^ approximately).

All interfacial tension experiments were carried out at pH 7, thus it is not surprising to see that peptides 1, 3 and 5 reached the equilibrium interfacial tension at lower values as compared to peptides 2, 4 and 6 (S3 Table). It has been previously reported [40–42] that interfacial activity of amphiphilic molecules and surfactants is mainly attributed to polar interactions provided that there is enough ionization at adequate pH conditions. In fact, the case of the indigenous surfactants presents in crude oil (e.g. asphaltenes, resins, and naphthenic acids) that stabilize emulsions (W/O and O/W) serves as an example to visualize what is happening with the peptides as they are also not strictly an amphiphilic molecule in the sense that a polar head and aliphatic tail cannot be truly distinguished. Instead, they have polar, aromatic and aliphatic regions [43,44] just like the peptides. For instance, asphaltenes tend to be acidic (i.e. there is a significant concentration of–COOH groups per asphaltene molecule) and their interfacial activity is mainly driven by H-bonding [45]. At high pH values, asphaltenes become highly surface-active causing a dramatic reduction of the interfacial tension (oil/water interface) whereas at neutral pH the response is almost negligible [46,47]. As shown in Table 1, the isoelectric point of peptides 1, 3 and 5 oscillates between pH 6.74 and pH 8.25, which means that these molecules are at their maximum interfacial activity. Furthermore, peptide 1 has a more amphiphilic orientation than peptides 3 and 5 because all the hydrophilic groups are located on one side of the molecule (Fig 4). This might allow a more organized adsorption layer at the liquid-liquid interface that minimizes the steric interactions. Such interactions were shown to be more pronounced in peptide 5, where the hydrophilic groups are distributed through the entire molecule (Fig 4). In contrast, as peptide 3 showed an intermediate distribution of hydrophilic groups, this could explain its intermediate value for the equilibrium interfacial tension.

On the other hand, the isoelectric point of peptides 2, 4 and 6 are located on the acidic region (between pH 4.21 and pH 5.83) which means that little interfacial activity is expected given that ionization of–COOH or–NH_2_ groups would be low. This is what the interfacial tension data of Fig 7 and S3 Table shows corroborating the hypothesis of the close relationship between ionization and interfacial activity. Furthermore, under unfavorable pH conditions comparing peptides 2, 4 and 6, peptide 4 exhibited the lowest equilibrium interfacial tension value. This is consistent with our previous observations as the distribution of hydrophilic groups is uneven (i.e. located in one side of the molecule) giving it a more amphiphilic character and more importantly, a favorable adsorption orientation that reduces steric interactions in the adsorbed layer.

Finally, combining DSC and DLS data revealed that the structure of peptide 5, which displayed two hydrophobic domains on opposite sides of the molecule, can be related to the variability in interfacial tension and its enthalpy contribution. This peptide was the one with the highest molecular weight, which also showed a lower variability of droplet size over time. Although a similar pattern was observed in the Gaussian peak distribution of peptide 6, this peptide reached the interface in a short period of time. This could be explained by the fact that peptide 6 is a smaller molecule that can easily diffuse through the components of crude oil.

It is important to mention that interfacial tension measurements were in partial agreement with the molecular dynamics. Although peptide 1 exhibited the highest ΔG_sol_ /SASA, ΔG_sol_ /MW, ΔG_Coulomb_/ ΔGV_dW_ values and dependency among preservation time, surfactant concentration, and crystallization phenomenon, we cannot safely conclude that the results of the molecular dynamics simulations can be related to the crystallization temperatures variations. Crystallization in emulsions stabilized with the peptides 1 and 5 was also influenced by both the peptide concentration and the preservation time. However, there is no experimental evidence that confirmed the outstanding performance of these peptides against the n-dodecane-water interface or the crude oil-NaCl (1M) interface.

Consequently, interactions at the oil-water interface need to be captured by more parameters during simulation because they are crucial to confer temporal stability to the emulsion. We found that due to nucleation (which leads to supercooled and supersaturated liquids) the DSC curves obtained during cooling and heating are quite different, and only the crystallization phenomenon can be used as a powerful tool to understand simultaneously the evolution of dynamic properties such as droplet size distribution and the thermodynamic implications of the interactions at the oil-water interface. Moreover, it is important to mention that other factors such as peptides secondary structures may be also influencing peptides assembly at interfaces. The relevance of studying the mechanism controlling surfactants structural changes at emulsion interfaces and how this affects emulsion stability and adsorption have been previously highlighted [48,49]. Our preliminary results regarding the secondary structure of the six rational designed peptides have revealed the presence of a large variety of structural motifs (S5 Fig). It is important to mention that future studies will be required to assess the relevance of these motifs on emulsion stability.

## Conclusions

In the present work, we have used Differential Scanning Calorimetry (DSC), Dynamic Light Scattering (DLS) and Interfacial Tension (IT) measurements to investigate the behavior of six synthetic rationally-designed peptides based on *E*.*coli*’s OmpA at the water-dodecane interface. Our data revealed that the emulsions stabilized with peptides 1, 2 and 5 displayed a correlation between the amount of surfactant and the crystallization temperature of water and n-dodecane. Besides, emulsions prepared with peptide 1 and 5, also showed a dependency between the preservation time and the crystallization temperatures. Interestingly, IT measurements allowed to divide the peptides into two different groups: i) uncharged or smaller peptides that reached the interface faster (peptides 1, 4 and 6) and ii) peptides that needed more time to be adsorb at the interface (peptides 2, 3 and 5), which are possibly charged or feature a structure without defined hydrophobic and hydrophilic moieties. Regardless of the mechanism by which the peptides reached the interface, in all the cases we observed that the crude oil–NaCl 1M interfacial tension was reduced.

Our data suggest that viable biodegradable surfactants of organic nature can be custom made depending on the application, and more importantly, the distribution of hydrophilic groups, responsible for the interfacial activity, can be easily fine-tuned. We strongly believe that our findings could impact the search of novel surfactants of biodegradable nature, which could have different applications in various industries such as cosmetics, food, environmental and petrochemical.

## Supporting information

S1 Fig Influence of peptide concentration on the crystallization temperature (°C) of water (A) and n-dodecane (B) in O/W emulsions for peptide 3 (circles), peptide 4 (rhombus), and peptide 6 (squares). (TIF)

S2 Fig Cooling thermograms for the six rationally designed peptides at 0.05%, 0.15% and 0.25% (w/v). The dashed lines represent the individual peaks after deconvolution.
(TIF)

S3 Fig Influence of the preservation time on the water crystallization temperature for peptide 2 (circles), 3 (rhombus) and 6 (squares). (TIF)

S4 Fig Enthalpy contribution of each Gaussian peak to the total crystallization enthalpy (including n-dodecane and water) for 0, 2, 4 hours of preservation time. (TIF)

S5 Fig FTIR spectrum, amide I band and the second derivative amide I spectrum for each rationally designed peptide. Water spectrum was digitally subtracted from all the FTIR spectra. (A) Peptide 1. The marked peaks correspond to β-sheet (1624 and 1638 cm^−1^), 310Helix (1660 cm^−1^) and β-Turn (1678 cm^−1^). (B) Peptide 2. The marked peaks correspond to β-sheet (1634 cm−1), random coil (1647 cm^−1^), α Helix (1654 cm^−1^), 310Helix (1663 cm^−1^) and β-Turn (1674 cm^−1^). (C) Peptide 3. The marked peaks correspond to β-sheet (1623, 1628, 1634 and 1639 cm^−1^), random coil (1646 and 1650 cm^−1^), α Helix (1655 cm^−1^), 310Helix (1660 cm^−1^) and β-Turn (1678 cm^−1^). (D) Peptide 4. The marked peaks correspond to β-sheet (1624, 1631 and 1637 cm−1), random coil (1647 cm−1), α Helix (1654 cm^−1^), 310Helix (1660 cm^−1^) and β-Turn (1678 cm^−1^). (E) Peptide 5. The marked peaks correspond to β-sheet (1634 and 1642 cm^−1^), random coil (1649 cm^−1^), α Helix (1654 cm^−1^), 310Helix (1664 cm^−1^) and β-Turn (1678 cm^−1^). (F) Peptide 6. The marked peaks correspond to β-sheet (1624, 1631, 1634, 1639, 1641, 1692 and 1695 cm^−1^), random coil (1649 cm^−1^), α Helix (1654 and 1657 cm^−1^), 310Helix (1661 and 1666 cm^−1^) and β-Turn (1668, 1674, 1678, 1680, 1684 and 1686 cm^−1^).
(TIF)

S1 Table Highest free energy change per molecular weight (ΔGsolv/MW) and solvent-accessible surface areas (SASAs) obtained from molecular dynamics simulations for the six synthesized peptides and OmpA. Peptides were ranked according to the ΔGsolv/SASA and ΔGsolv/MW.
(DOCX)

S2 Table Characterization of the crude oil employed to perform the interfacial tension measurements. (DOCX)

S3 Table Reduction of interfacial tension by the six peptides at a final concentration of 550 ppm. (DOCX)
