# Supplementary figures and images for: Insights into the behavior of six rationally designed peptides based on Escherichia coli’s OmpA at the water-dodecane interface
Source: PLoS One. 2019 Oct 10;14(10):e0223670. doi: 10.1371/journal.pone.0223670 (PMC6786535; doi:10.1371/journal.pone.0223670)

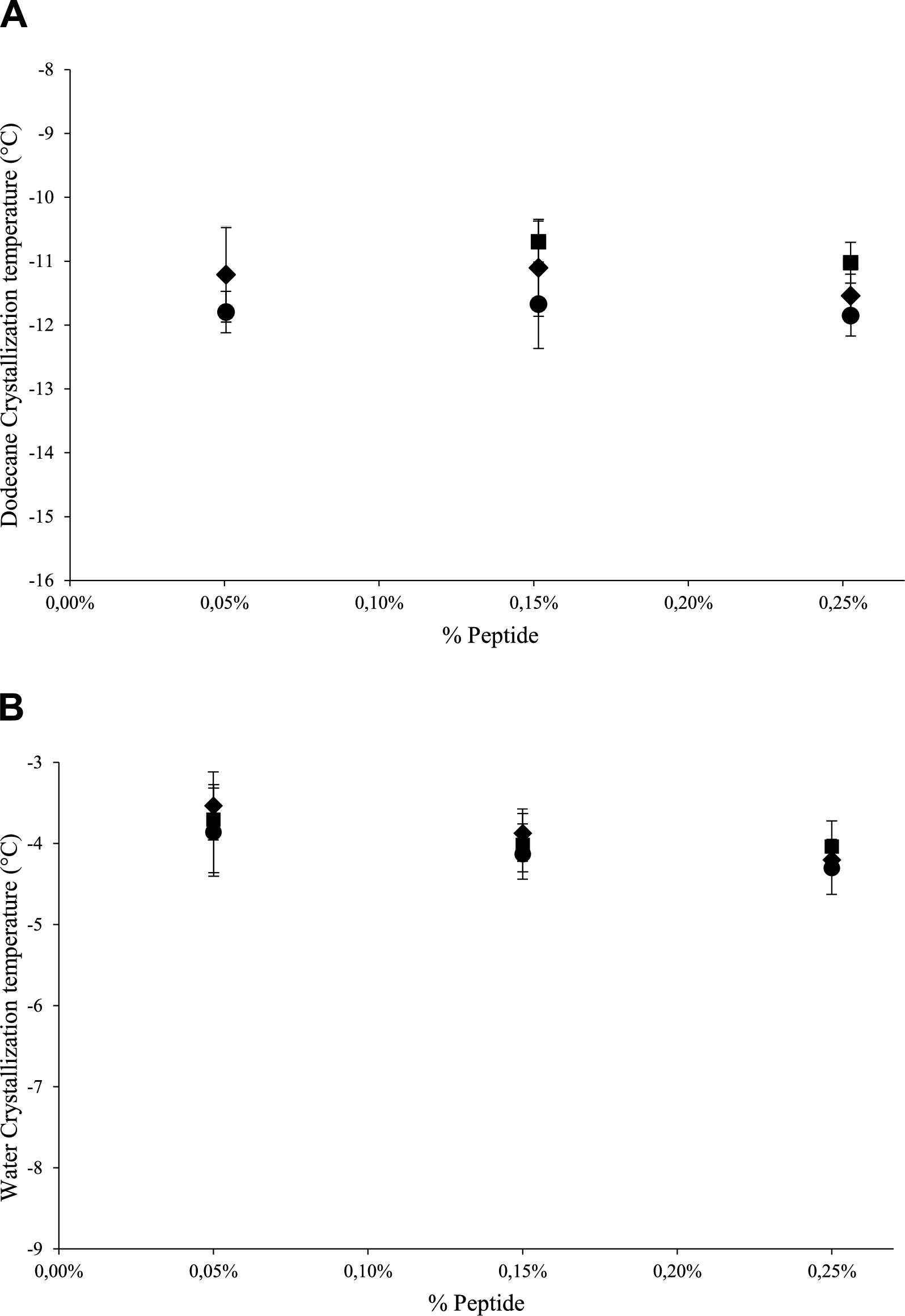

Supplement: S1 Fig — (TIF) [file pone.0223670.s001.tif]

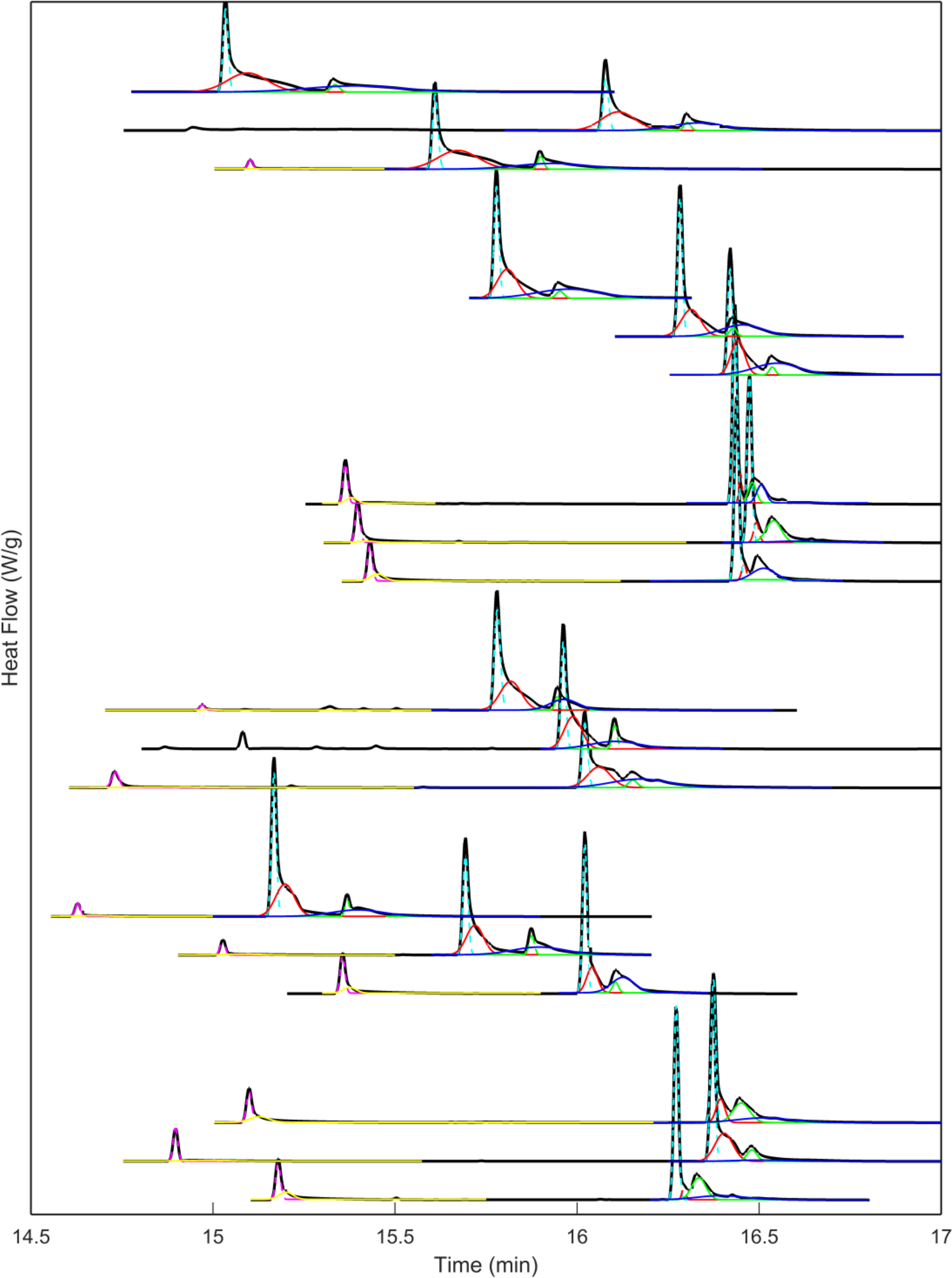

Supplement: S2 Fig — The dashed lines represent the individual peaks after deconvolution. (TIF) [file pone.0223670.s002.tif]

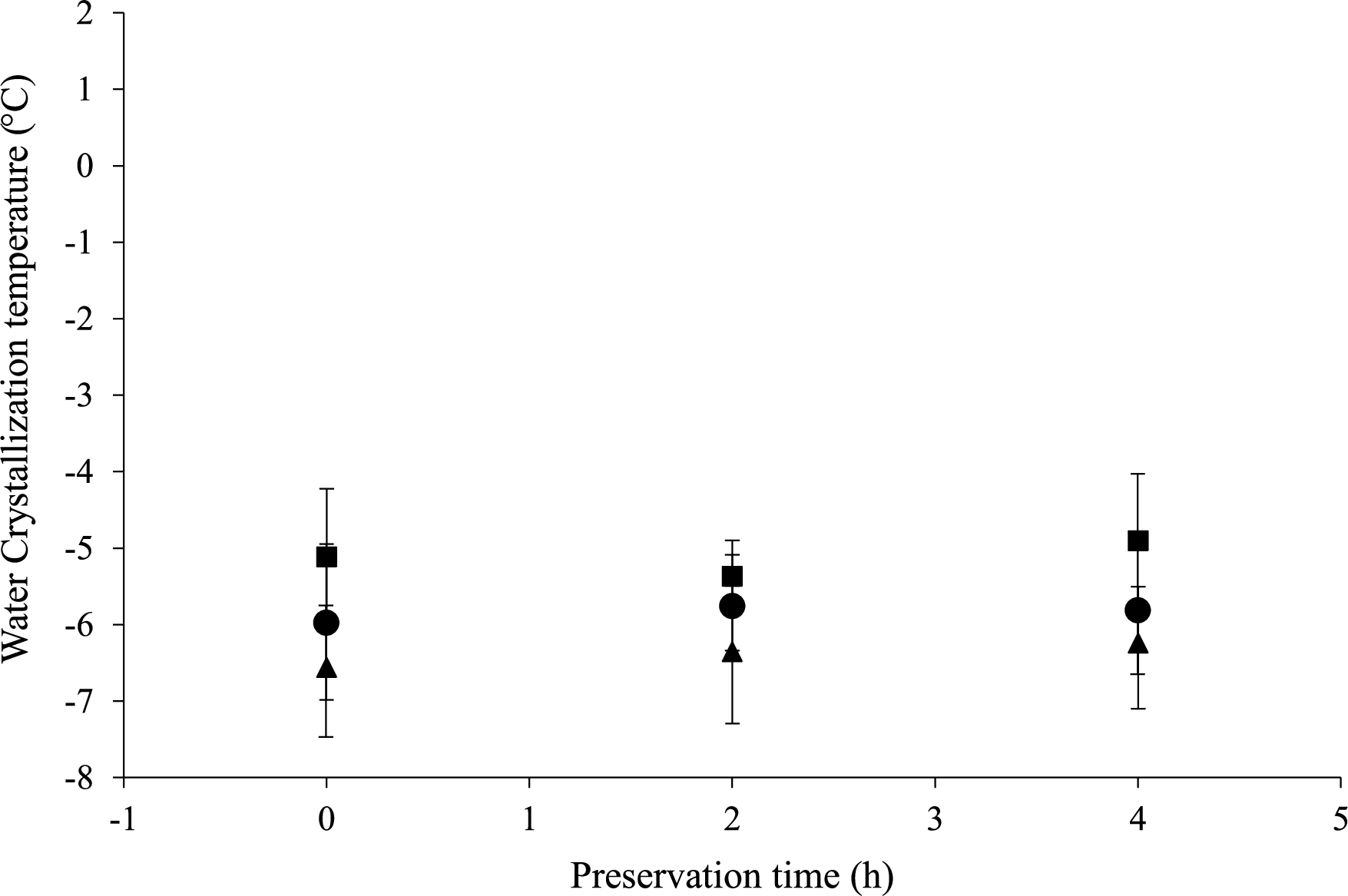

Supplement: S3 Fig — (TIF) [file pone.0223670.s003.tif]

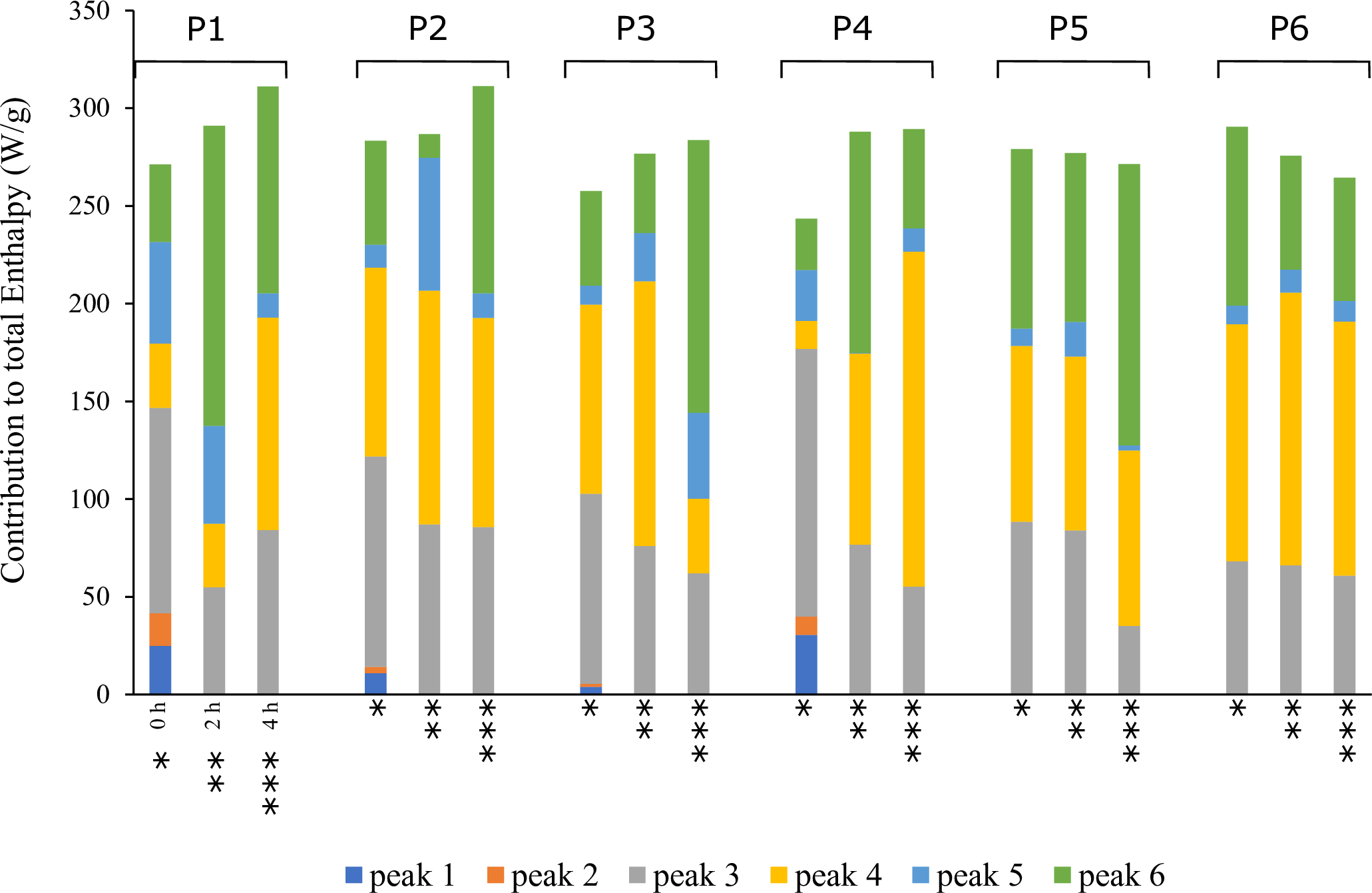

Supplement: S4 Fig — (TIF) [file pone.0223670.s004.tif]

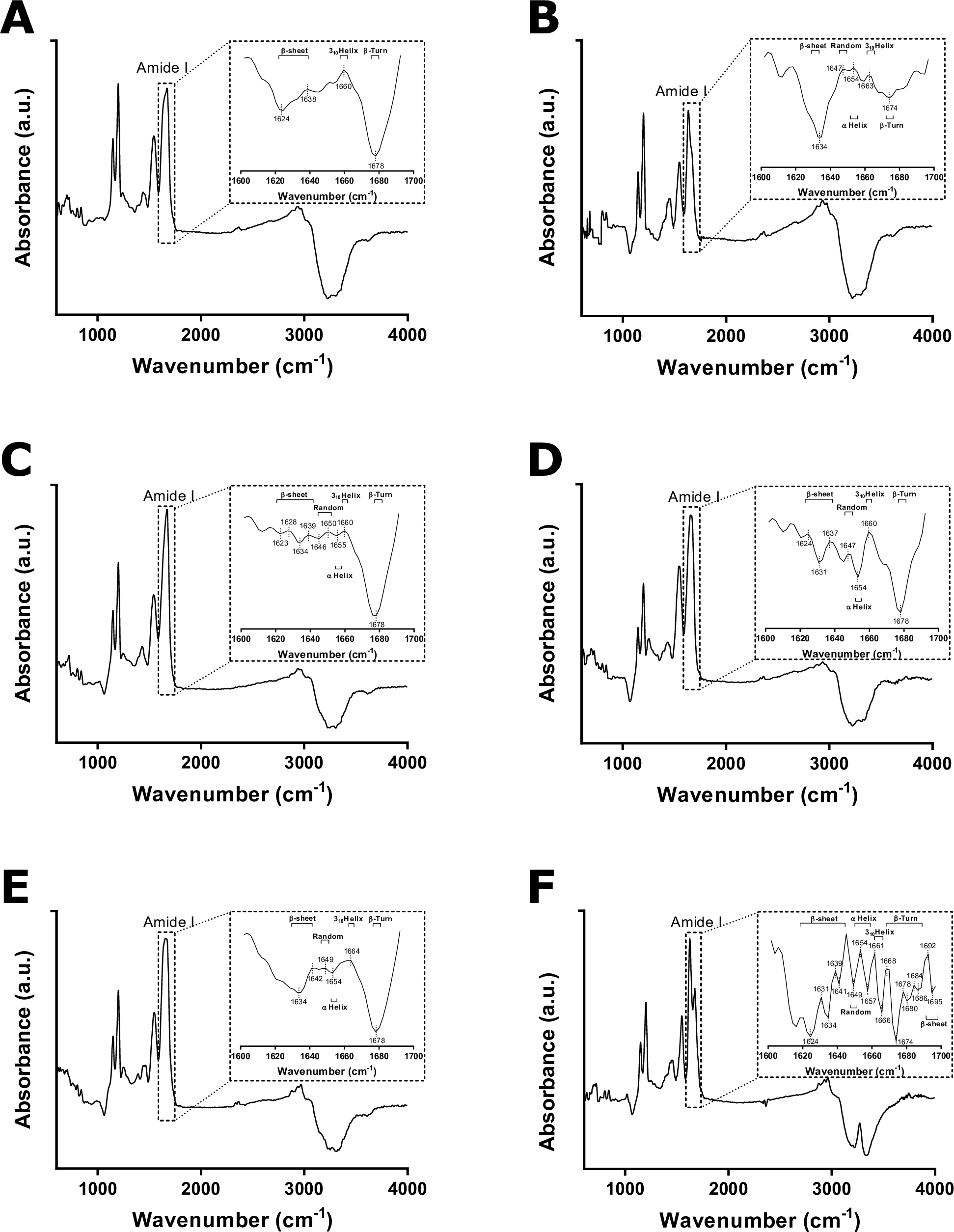

Supplement: S5 Fig — Water spectrum was digitally subtracted from all the FTIR spectra. (A) Peptide 1. The marked peaks correspond to β-sheet (1624 and 1638 cm−1), 310Helix (1660 cm−1) and β-Turn (1678 cm−1). (B) Peptide 2. The marked peaks correspond to β-sheet (1634 cm−1), random coil (1647 cm−1), α Helix (1654 cm−1), 310Helix (1663 cm−1) and β-Turn (1674 cm−1). (C) Peptide 3. The marked peaks correspond to β-sheet (1623, 1628, 1634 and 1639 cm−1), random coil (1646 and 1650 cm−1), α Helix (1655 cm−1), 310Helix (1660 cm−1) and β-Turn (1678 cm−1). (D) Peptide 4. The marked peaks correspond to β-sheet (1624, 1631 and 1637 cm−1), random coil (1647 cm−1), α Helix (1654 cm−1), 310Helix (1660 cm−1) and β-Turn (1678 cm−1). (E) Peptide 5. The marked peaks correspond to β-sheet (1634 and 1642 cm−1), random coil (1649 cm−1), α Helix (1654 cm−1), 310Helix (1664 cm−1) and β-Turn (1678 cm−1). (F) Peptide 6. The marked peaks correspond to β-sheet (1624, 1631, 1634, 1639, 1641, 1692 and 1695 cm−1), random coil (1649 cm−1), α Helix (1654 and 1657 cm−1), 310Helix (1661 and 1666 cm−1) and β-Turn (1668, 1674, 1678, 1680, 1684 and 1686 cm−1). (TIF) [file pone.0223670.s005.tif]
