# Supplementary material for: Insights into the behavior of six rationally designed peptides based on Escherichia coli’s OmpA at the water-dodecane interface
Source: PLoS One. 2019 Oct 10;14(10):e0223670. doi: 10.1371/journal.pone.0223670 (PMC6786535; doi:10.1371/journal.pone.0223670)
Supplement: S1 Table — Peptides were ranked according to the ΔGsolv/SASA and ΔGsolv/MW. (DOCX) [file pone.0223670.s006.docx]

**S1 Table.** **Highest free energy change per molecular weight (ΔG_solv_/MW) and solvent-accessible surface areas (SASAs) obtained from molecular dynamics simulations for the six synthesized peptides and OmpA.** Peptides were ranked according to the ΔG_solv_/SASA and ΔGsolv/MW.

| Peptide | ΔGsolv/SASA | Peptide | ΔGsolv/MW |
| --- | --- | --- | --- |
| P1 (GKNHDTGVSPVFA) | -60.65 | **P2** | -0.73 |
| P2 (DPKDGSVVVL) | -54.45 | **P1** | -0.69 |
| P3 (TGNTCDNVKQR) | -50.32 | **P5** | -0.54 |
| P4 (THENQLGAGAFG) | -46.77 | **P3** | -0.54 |
| P5 (QRAALIDCLAPDRRV) | -45.39 | **P4** | -0.49 |
| OmpA | -41.1 | **P6** | -0.44 |
| 6 (QRAALIDCLA) | -40.81 | **OmpA** | -0.24 |
