# Supplementary material for: Insights into the behavior of six rationally designed peptides based on Escherichia coli’s OmpA at the water-dodecane interface
Source: PLoS One. 2019 Oct 10;14(10):e0223670. doi: 10.1371/journal.pone.0223670 (PMC6786535; doi:10.1371/journal.pone.0223670)
Supplement: S2 Table — (DOCX) [file pone.0223670.s007.docx]

**S2 Table.** **Characterization of the crude oil employed to perform the interfacial tension measurements.**

| Density (g/cm3) | 0.986 |
| --- | --- |
| °Api | 12 |
| $\boldsymbol{\mu}$ [Pa$\boldsymbol{\cdot}$s] @ 25°C | 113.1 |
| pH @ 25°C | 6.21 |
| Water Content (% v/v) | 10 |
| Saturate (%) | 31.22 |
| Aromatic (%) | 38.1 |
| Resin (%) | 22.35 |
| Asfalten (%) | 8.33 |
