# Supplementary material for: Insights into the behavior of six rationally designed peptides based on Escherichia coli’s OmpA at the water-dodecane interface
Source: PLoS One. 2019 Oct 10;14(10):e0223670. doi: 10.1371/journal.pone.0223670 (PMC6786535; doi:10.1371/journal.pone.0223670)
Supplement: S3 Table — (DOCX) [file pone.0223670.s008.docx]

**S3 Table.** **Reduction of interfacial tension by the six peptides at a final concentration of 550 ppm.**

|  | Interfacial tension (mN/m) |
| --- | --- |
| Negative Control | 44.9±4.45 |
| Tween® 20 | 35.8±0.877 |
| P1 (GKNHDTGVSPVFA) | 30.1±1.342 |
| P2 (DPKDGSVVVL) | 42.5±2.876 |
| P3 (TGNTCDNVKQR) | 31.6±0.532 |
| P4 (THENQLGAGAFG) | 38.3±0.324 |
| P5 (QRAALIDCLAPDRRV) | 36.6±9.456 |
| P6 (QRAALIDCLA) | 39.1±3.245 |
